# Altered Inflammatory Protein Release by Vascular Smooth Muscle Cells in 3D-Culture

**DOI:** 10.3390/ijms27198567

**Published:** 2026-09-25

**Authors:** Karin Fransén, Enna Lokmic, Liisa Vasemägi, Madelene Lindkvist

**Affiliations:** 1 Cardiovascular Research Centre, Faculty of Medicine and Health, Örebro University, 701 82 Örebro, Sweden; karin.h.franzen@oru.se (K.F.);; 2 School of Medical Sciences, Faculty of Medicine and Health, Örebro University, 701 82 Örebro, Sweden

**Keywords:** aortic smooth muscle cell, spheroids, inflammation, proteomics, inflammasome

## Abstract

Aortic smooth muscle cells (AoSMCs) are widely used in cardiovascular research for studies on inflammatory processes in atherosclerotic lesions. Usually, AoSMCs are cultured in two-dimensional (2D) cell culture models, although limitations exist compared to the three-dimensional (3D) environment, and differences between 2D and 3D conditions on inflammatory markers have not been explored. This study aims to elucidate differences in inflammatory biomarkers in 2D versus 3D cultured AoSMCs. After inflammatory treatment of 2D and 3D cultured cells, protein release and gene expression were measured by Olink proteomics/enzyme-linked immunosorbent assay and quantitative real-time polymerase chain reaction, respectively. The 2D cultured cells showed generally higher release of inflammatory proteins, such as C-C motif chemokine 20 (CCL20), transforming growth factor alpha (TGF-α) and TNF superfamily member 12 (TWEAK), compared to the 3D model. In contrast, more matrix metallopeptidase-10 (MMP-10) and interleukin-8 (IL-8) were released from 3D cultured cells compared to 2D cultured cells. Elevated release of interleukin-1β in addition to transcriptional activation of inflammasome components was also observed in the 2D condition compared to the 3D condition. In conclusion, release of inflammatory proteins differs depending on the cell culture model, where 2D cultured AoSMCs release higher levels of several pro-inflammatory proteins compared to 3D cultured AoSMCs.

## 1. Introduction

Cardiovascular diseases (CVDs) are the global leading cause of death and represent an estimated frequency of more than 19.4 million deaths every year [1].

Atherosclerosis is the underlying factor for many CVDs and is characterized by lipid depositions in parts of the arterial wall, adhesion and infiltration of mononuclear cells followed by inflammatory processes, proliferation of smooth muscle cells and formation of fibrous tissue, eventually leading to an ischemic event [2]. It is well-known that inflammation is a hallmark of atherosclerosis and a broad range of pro-inflammatory mediators, including interleukin (IL)-1β, produced by the NLR family pyrin domain containing 3 (NLRP3) inflammasome, and tumor necrosis factor (TNF) being released from immune-competent cells and other cell types in the lesion, including smooth muscle cells [3,4]. Targeting inflammatory biomarkers in atherosclerosis is a future therapeutic option in CVDs, and IL-1β has been pointed out as a putative future target in the previous Canakinumab Anti-inflammatory Thrombosis Outcome Study (CANTOS) trial [5].

However, implementation of novel drug candidates in the clinic against vascular inflammation requires substantial biomedical and clinical characterization. By in vitro monolayer two-dimensional (2D) cell culture models with aortic smooth muscle cells (AoSMCs) or endothelial cells, several recent advances have been accomplished [6]. The 2D cell culturing system is usually based on surface adherence to provide mechanical support [7]. There are however limitations to depicting the complex cellular processes in atherosclerosis with 2D cell cultures, and it has been argued that cells require a three-dimensional (3D) environment, involving adhesion to the extracellular matrix (ECM) and neighboring cells to form physiologically relevant tissue-like structures that resemble in vivo conditions [8,9]. Several different methods have been developed to generate 3D cell cultures, involving scaffold-based and scaffold-free cell cultures [10]. The scaffold-based techniques focus on building an ECM-like environment, providing a physical structure such as hydrogel, paper-based and fiber-based scaffolds. In contrast, scaffold-free 3D methods rely on cell-to-cell adhesion to form spheroids and their own ECM, without the pre-made physical structure, and include techniques like low-adhesion culture plates with specialized anti-adhesive surface coatings and the hanging drop method [11,12].

Studies comparing the inflammatory response between 2D and 3D cell cultures, specifically on AoSMCs, remain limited and further research comparing inflammatory markers in 2D versus 3D models could be of importance for establishing a broader understanding of the progression of the inflammatory process in atherosclerosis. The aim of this study is to compare 2D and 3D cell cultures, focusing on expression and release of inflammatory markers by proteomics and gene expression analysis on candidate proteins and corresponding genes, including the NLRP3 inflammasome.

## 2. Results

### 2.1. Aortic Smooth Muscle Cells in 3D Cell Culture

Culture of AoSMCs in U-bottomed wells induced the formation of one single spheroid 3D structure per well (Figure 1A–D). The spheroids formed into a globular structure within a few hours and were stable enough to not decompose by handling like media exchange.

### 2.2. Proteomic Evaluation of Protein Release from 2D and 3D Cell Cultures

Levels of 92 different inflammatory proteins released from the AoSMCs were measured by the Olink^®^ (Waltham, MA, USA) proteomics Inflammation panel. In total, 47 of 92 protein assays were expressed in more than 50% of the samples and were therefore included in further analysis (for the full list, see Supplementary Table S1). Overall, significant differences in protein expression between 2D and 3D cell cultures were observed in 16 of the 47 protein assays (*p* < 0.05) (Figure 2 and Figure 3). Out of these 16 differentially expressed proteins, release of 8 proteins was altered between 2D and 3D cell culture models across all treatment conditions (Figure 2A–H). Protein release was significantly upregulated in 2D cell culture compared to 3D cell culture for eukaryotic translation initiation factor 4E-binding protein 1 (4E-BP1, Figure 2A), adenosine deaminase (ADA, Figure 2B), eotaxin (CCL11, Figure 2C), CUB domain-containing protein 1 (CDCP1, Figure 2D), interleukin-1 alpha (IL-1α, Figure 2E), and monocyte chemoattractant protein-4 (MCP-4, Figure 2G). In contrast, IL-8 (Figure 2F) and matrix metallopeptidase 10 (MMP-10, Figure 2H) were significantly upregulated in 3D cell culture compared to the 2D cell culture model.

When comparing the response of inflammatory treatments to the untreated controls within each cell model of 2D and 3D cell cultures, 8 out of the 16 differentially expressed proteins between models also had significant alterations in protein release in response to treatments (Figure 3A–H). These 8 proteins were upregulated in 2D cell culture compared to 3D cell culture. Release of C-C motif chemokine 20 (CCL20, Figure 3A) was significantly upregulated by both LPS treatment alone and in combination with ATP treatment for both 2D and 3D cell cultures compared to their respective untreated control. Treatment with LPS and ATP upregulated protein release of TNF superfamily member 12 (TWEAK, Figure 3H) in both 2D and 3D cultures, and in 2D cell cultures, fibroblast growth factor 5 (FGF-5, Figure 3C) was also significantly induced. Protein release of Delta and Notch-like epidermal growth factor-related receptor (DNER, Figure 3B), FGF-21 (Figure 3D), glial cell-derived neurotrophic factor (GDNF, Figure 3E), MCP-3 (Figure 3F) and transforming growth factor alpha (TGFα, Figure 3G) were significantly up- or downregulated compared to their untreated controls in either the 2D or 3D cell culture model by LPS alone or in combination with ATP treatment. Despite statistical significance for these proteins, there were only minor changes in values, suggesting the limited physiological relevance of these alterations.

The proteomic analysis also revealed 4 proteins that had altered release in response to LPS and ATP treatment without any significant differences between 2D and 3D cell culture models. These proteins were C-X-C motif chemokine 10 (CXCL10, Figure S1A), transforming growth factor beta-1 proprotein (LAP TGF-β-1, Figure S1B), neurturin (NRTN, Figure S1C) and urokinase-type plasminogen activator (uPA, Figure S1D).

### 2.3. Alterations in Gene Expression Between 2D and 3D Cell Cultures

To study alterations in mRNA expression between 2D and 3D cell cultures, we selected 5 proteins with increased protein release in 2D compared to 3D cell cultures which also were significantly altered by inflammatory treatment compared to the untreated control. In addition, MMP-10 was also selected since increased protein expression was observed in 3D cell cultures compared to 2D cell cultures (Figure 4A–F). The gene expression was significantly increased in 2D cell cultures compared to 3D cell cultures for *FGF5* (Figure 4B) and *TGFA* (Figure 4E) in a similar pattern as their corresponding protein expression. In addition, gene expression of *TGFA* was also increased in 2D cell culture in response to LPS and ATP treatment compared to untreated control after 24 h. No significant alterations in gene expression between 2D and 3D cell cultures or inflammatory treatments were detected for *CCL20* (Figure 4A), *GDNF* (Figure 4C) or *TNFSF12* (Figure 4F, mRNA for TWEAK protein). Gene expression for *MMP10* showed a trend towards increased mRNA levels in 3D cell cultures compared to 2D cell cultures (Figure 4D) similar to the corresponding protein expression; however, it was not statistically significant due to the large variation between experiments. To control for hypoxia and cell death in the 3D model, *HIF1A* (Figure 4G) and *TP53* (Figure 4H) were analyzed after 24 h treatment and showed no indications of increased gene expression of the hypoxia or cell death markers in the 3D cell cultures compared to the 2D cell cultures. Expression of *HIF1A* in the untreated cells was even slightly reduced in the 3D model compared to the 2D model (Figure 4G).

### 2.4. IL-1β and NLRP3 Inflammasome Components in 2D and 3D Cell Cultures

Since IL-1β is central in the pathogenesis of CVDs, we investigated if the cytokine or the NLRP3 inflammasome were altered in 2D versus 3D cell culture models. However, IL-1β is not included in the Olink Inflammation panel, hence the secreted levels of this protein from the 2D and 3D cell cultures were measured using ELISA. Release of IL-1β was increased in 2D cell culture compared to 3D cell culture (Figure 5A) while there were no significant alterations in gene expression (Figure 5B). Gene expression of *NLRP3* was significantly increased in 2D cell culture compared to 3D cell culture (Figure 5C) while *CASP1* had a similar trend (Figure 5D); however, it was not statistically significant.

## 3. Discussion

Inflammation is a key factor in a wide range of diseases, and the process involves several layers of cells forming multiple tissue sheets [13,14]. Monolayered 2D models are currently the most widely used for basic cell culture research, although studies on 3D models allow the study of interactions between cells and their environment [6,15]. In the present study, we investigated the release of central inflammatory markers from AoSMCs with Olink proteomics and compared the inflammatory markers between 2D and 3D cell cultures since the inflammatory response may be affected by differences in cell culture conditions [6,16,17]. The AoSMCs were selected as a physiologically relevant model since smooth muscle cells represent the most frequent cell type in atherosclerotic lesions forming three-dimensional layers in vivo [18]. Furthermore, AoSMCs are a cell type commonly used for studies on vascular inflammation [18], and the frequent usage of these cells as a model for CVDs justifies descriptive studies on differences between 2D and 3D cell culturing. The aim of the study was to investigate inflammatory markers induced by LPS or LPS + ATP to generate a general pro-inflammatory milieu or inflammasome activation. Our data showed reduced release of several proteins that have been observed to be involved in central aspects of atherosclerosis, such as chemotaxis, proliferation, survival, metabolism and activation of leukocytes [19,20,21]. Among these, proteins like 4E-BP1, ADA, CCL11, CDCP1, IL-1α, MCP-4 and IL-1β were found to be increased in the 2D cell model compared to the 3D cell model across treatment conditions. Furthermore, an additional 8 proteins, CCL20, DNER, FGF-5, FGF-21, GDNF, MCP-3, TGF-α and TWEAK were all elevated in the 2D model compared to the 3D model also after significant response to inflammatory treatment. However, as smooth muscle cells are not primarily inflammatory cells, it can be challenging to study inflammasome activation in AoSMCs [22], which could explain the relatively low response to LPS treatments in both of our cell culture models.

A previous study, where NF-κB signaling was inhibited in 3D cultured cells, suggested that 3D cultured spheroids of vascular smooth muscle cells transform their gene expression pattern into a more resting phenotype compared to flat 2D cultured cells [23]. This mechanism could represent one possible underlying reason for the generally lower levels of protein release in our 3D model compared to the conventional 2D culture model. On the transcriptional level, we only observed elevated levels of *FGF5* and *TGFA* in the 2D model compared to the 3D model, although nonsignificant trends resembling the protein data for GDNF and MMP-10 were observed. In the present study, we selected one single time-point of 24 h of incubation to study since we and other studies on protein expression have selected this time-point [24,25]. We also studied gene expression at 24 h, which indicated a variability in expression between mRNA and protein levels. This observed variation in expression probably reflects the fact that gene transcription is an earlier event than translation [26]. However, detailed evaluation of the structural growth of the spheroids and evaluation of gene expression at shorter time-points is beyond the focus of the present study and thereby remains to be elucidated by future investigations. In contrast to all markers with reduced protein release in the 3D condition, we observed an increased MMP-10 release in the 3D model compared to the 2D model. The MMP-10 protein is a proteolytic enzyme that breaks down elastin, fibronectin, laminin and several types of collagens and is involved in ECM remodeling [27]. Furthermore, MMP-10 has been identified as a profibrotic marker and associated with vascular remodeling [28,29]. Elevated levels in the 3D condition could therefore be a result of altered architecture and organization compared to the 2D model. Our data are supported in the literature, which has shown increased MMP-10 levels in 3D culture compared to 2D models, although in other cell types [30]. A possible explanation for the increased MMP-10 release in the 3D model might be due to alterations in the surrounding ECM compared to the 2D model. Previous studies have shown that MMP-10 acts as a modulator of collagen deposition and tissue stiffness, which might reflect the different growth patterns in the two conditions [31]. Previously, stiffness of the ECM was observed to lead to increased mechanotransduction in 3D models in a way that could not be observed in 2D models [32]. How mechanical cues from the ECM are transduced in the 3D context is however not yet fully understood. However, ECM-embedment of cells in 3D models could contribute to an altered mechanotransduction, which in turn could affect secretion, activation or transcription of proteins [33]. This could be one putative contributing factor to the differences we noticed between 2D and 3D cultured cells.

In the present study, we also investigated the gene expression of NLRP3 inflammasome components and release of IL-1β as a marker for NLRP3 inflammasome assembly in the 2D and 3D models of AoSMCs. The NLRP3 inflammasome assembly is a two-step process; the priming step via activation of toll-like receptor (TLR) 4/6 by danger associated molecular patterns (DAMPs) or pathogen associated molecular patterns (PAMPs) followed by NF-κB activation and elevated gene expression of *IL1B* and *NLRP3* that induce the production of pro-IL-1β. The priming step is followed by the activation step, which may consist of various signals such as ATP, K^+^ efflux, cholesterol crystals and lysosomal damage or reactive oxygen species production [34]. The precursor of IL-1β is cleaved to its’ active form by caspase-1 by the NLRP3 inflammasome. In the present study, we detected higher *NLRP3* gene expression and IL-1β release in the 2D model compared to the 3D model across treatment groups. This could indicate a more easily induced NLRP3 inflammasome in the 2D model compared to the 3D model. However, we could not prove any difference in *IL1B* expression after 24 h, possibly due to the activation of *IL1B* gene expression at an earlier time point. Also, our proteomics data indicate that 2D cell culturing compared to 3D cell culturing is characterized by an enhanced inflammatory level.

Previous studies have indicated that 2D cultured smooth muscle cells tend to form a heterogenous way of growth, with overlapping cell layers with hills and nodules and even microfibrils after a long culture time, which may suggest that 2D cultured AoSMCs strive for a 3D growth pattern [35,36]. This data therefore suggest that 3D growth patterns may be considered as a more pre-programmed way of growth for the AoSMCs.

As with all 3D culture models, it is challenging to control factors such as differences in exposure to the treatments, nutrients and oxygen availability between central and peripheral cells in the spheroid [37], factors that could affect experiments in different cellular models. Since the cellular structure and surrounding environment have important roles in regulating the cellular response, including gene expression and cytokine secretion, the environment in which the cells are cultivated could lead to an altered response to the inflammatory stimuli in different cell culture models due to the nature of the model. To assess whether hypoxia and cell death within the 3D model could have influenced the generally lower release of inflammatory proteins, gene expression of *HIF1A* and *TP53* was analyzed in both cell culture models. Our data show no indications of increased hypoxia or cell death in the 3D model compared to the 2D model; on the contrary, we noticed a small but significant reduction in *HIF1A* expression in the untreated 3D model compared to the untreated 2D model. Since expression of both hypoxia and apoptosis markers is low in all treatment conditions and both cell culture models, we do not expect this to have affected the results in this study. This supports our hypothesis that our experimental design comes with a low risk of hypoxia and apoptosis. We cannot exclude that other 3D models may result in other conclusions regarding these specific markers.

Although there may be several reasons for 3D cultured cells having mainly lower expression of inflammatory biomarkers compared to cells in the 2D model, the focus of the present study is solely descriptive, and the mechanistic factors remain to be elucidated in future investigations. In general, the 3D models are assumed to provide a bridge between 2D in vitro studies and in vivo studies [6]. Our data contributes to the knowledge that protein release cannot be directly translated between the two different models. The findings of the present study suggest that the two models differ in key variables central to regulation of the cellular environment and cell–cell contact.

In addition to the challenges of 3D models stated above, the present study has some limitations to take into consideration while interpreting the results. Except for utilization of a single time point for our experiments, usage of AoSMCs from a single donor could affect the generalizability of our data. We can therefore not exclude alternate results from other donors and studies of protein expressions at other time points. Another limitation is that we only know the number of seeded cells in the two different models. However, we utilized a relatively short time point of 24 h to limit the risk of central hypoxic conditions in the spheroids [38], and in this design, the 2D cultured cells showed equal or higher expression of hypoxic marker *HIF1A* compared to the 3D cultured cells. Future studies could be improved by also applying normalization of protein secretion to cell numbers after treatments. Regarding the low sample size, which is a common in vitro design, the data obtained should be considered exploratory. The results from the present study may therefore restrict the overall physiological interpretation, although it contributes to valuable knowledge on the variability between 2D and 3D cell culture models. Due to these limitations, our data should therefore be recognized as guidance for further research in this field. Collectively, our results show that there is broad variation in protein release of inflammatory markers between 2D and 3D cell culturing conditions, which suggests that 2D cultured cells in general have an elevated release of several inflammatory proteins, such as CCL20, TGF-α and TWEAK. In contrast, MMP-10 was released in lower concentrations in 2D compared to 3D cell cultures. Our data also highlight elevated IL-1β levels in the 2D cultured cells compared to the 3D model, which may indicate elevated NLRP3 inflammasome responsiveness in the 2D condition. This research contributes to the important knowledge that protein release cannot be directly translated between the two different cellular models. Understanding how inflammatory biomarkers act in the multilayered cells of the atherosclerotic lesion is of importance for advancement in the research of the disease and development of future therapeutic strategies.

## 4. Materials and Methods

### 4.1. Cell Culture

Human aortic smooth muscle cells (AoSMCs) (Thermo Fisher Scientific, Waltham, MA, USA) were cultured in Gibco^TM^ Human Vascular Smooth Muscle Cell Basal Medium, supplemented with Gibco^TM^ Smooth Muscle Growth Supplement (SMGS; final concentration of 4.9% fetal bovine serum, 2 ng/mL human basic fibroblast growth factor, 0.5 ng/mL human epidermal growth factor, 5 ng/mL heparin, 0.01 μg/mL recombinant human insulin-like growth factor-I, 0.2 μg/mL BSA) and 0.1 U/mL Penicillin + 100 ng/mL streptomycin (PEST, Gibco/Thermo Fisher Scientific, Waltham, MA, USA) in 75 cm^2^ culture flasks and incubated in 5% CO_2_ at +37 °C. The medium was changed every 48–72 h. Cells between passages 8 and 10 were used for experiments. Cells were cultured in Nunclon^TM^ Sphera^TM^ 96-well U-bottom plates (Thermo Fisher Scientific, Waltham, MA, USA) for 3D cell culture models. These round-bottomed, low-adherence well plates have a specific surface coating that forces the cells to repel the walls of the plate, supporting the cultured cells to form spheroids or organoids. The AoSMCs were seeded at a concentration of 1.0–1.5 × 10^4^ cells per well. In parallel with the 3D cultures, cells from the same flask were seeded at the same density (1.0–1.5 × 10^4^ cells per well) in conventional cell culture plates to create corresponding 2D cultures for each experiment. Both 2D and 3D cultures and experiments used 100 µL complete medium per well.

### 4.2. Imaging

Cells cultured into a 3D model of a spheroid were visualized on an EVOS M5000 microscope (Thermo Fisher Scientific, Waltham, MA, USA). Cell culture media was removed, and cells were fixed for 15 min and then permeabilized for 15 min, before they were washed in PBS. Spheroids were stained with smooth muscle cell marker F-actin (anti-human smooth muscle actin; M0851, DakoCytomation Denmark A/S, Glostrup, Denmark; 1:150) and nuclei-staining by DAPI (4′,6-diamidino-2-phenylindole; 5 μg/mL, Thermo Fisher Scientific, Waltham, MA, USA) for 10 min.

### 4.3. Treatments

To trigger a general inflammatory response, cells were treated with LPS (1 µg/mL) alone [39] or in combination with ATP (5 mM) to induce inflammasome activation [40]. Treatment with LPS occurred for 24 h, and ATP was added for the last hour of incubation. Cell culture media and cells were collected and stored at −80 °C until further analysis.

### 4.4. Proteomics by Olink Inflammation Panel

To analyze proteins that were released from cells of the 2D and 3D cultures, the culture medium from 4 independent experiments were analyzed by Olink Proteomics (Uppsala, Sweden) with the Olink Target 96 Inflammation panel. The panel contained 92 different biomarkers for inflammation (for the full list, see Supplementary Table S1). Olink uses Proximity Extension Assay (PEA) with 2 antibodies per target antigen that are marked with DNA barcodes. When the 2 antibodies bind to the same target protein in the cell medium solution, it enables DNA annealing and DNA extension, followed by amplification of the DNA and measurement by quantitative polymerase chain reaction (qPCR). The protein analysis is presented as normalized protein expression levels (NPX), where cycle threshold values are normalized for extension and interpolate control for accurate comparison of protein expression. Assays with over 50% missing data were excluded from further analysis.

### 4.5. RNA Extraction, cDNA Preparation, and Quantitative Real-Time PCR

An E.Z.N.A^®^ RNA extraction kit I (Omega Bio-tek, Norcross, GA, USA) was used according to the manufacturer’s instructions for total RNA extraction of AoSMCs from 3 independent experiments. Within each experiment, cells were pooled from 4 wells with the same experimental conditions to obtain a sufficient amount of material for cDNA synthesis.

A High-Capacity cDNA Reverse Transcription Kit (Thermo Fisher Scientific, Waltham, MA, USA) was used according to the manufacturer’s instructions for cDNA synthesis. An amount of 0.05 μg of total RNA was used per reaction. The cDNA samples were stored at −20 °C for further analysis. Gene expression was analyzed using TaqMan universal PCR master mix, TaqMan primers and probes in a QuantStudio 7 Flex real-time PCR system (Thermo Fisher Scientific, Waltham, MA, USA) according to the manufacturer’s instructions. The thermal cycle was initialized at 95 °C for 20 s, followed by 40 cycles of 95 °C for 1 s and 60 °C for 20 s. We selected proteins from the proteomic evaluation with both relevant differences between models and to untreated controls to assess gene expression; the probes used were *CCL20* (Hs 00355476), *FGF5* (Hs 03676587), *GDNF* (Hs 01931883), *MMP10* (Hs 00233987), *TGFA* (Hs 00608187) and *TNFSF12* (Hs 00387540). For quality control, gene expression of *HIF1A* (Hs00153153) and *TP53* (Hs 01034249) was analyzed. The probes used to detect inflammasome involvement were *IL1B* (Hs 01555410), *CASP1* (Hs 00354836) and *NLRP3* (Hs 00918082). Quantity values were normalized by the housekeeping gene peptidyl-prolyl-cis-trans isomerase B (*PPIB,* Hs 00168719). All probes were from Thermo Fisher Scientific, Waltham, MA, USA. 

### 4.6. Enzyme- Linked Immunosorbent Assay (ELISA)

To measure the levels of IL-1β in the cell medium from 5 independent experiments of 2D and 3D cultures, a sandwich ELISA kit for IL-1β (DY201, DuoSET^®^, R&D systems, Bio-techne Ltd, Abingdon, UK) was used according to the manufacturer’s instructions. The optical density of each well was determined using a BioTek Epoch 2 microplate reader (BioTek, Winooski, VT, USA) set to 450 nm with 540 nm correction, and concentrations of IL-1β (pg/mL) were calculated from the standard curve.

### 4.7. Statistics

Statistical analysis was conducted by using the program GraphPad Prism 10.4.2. To assess statistical differences in protein release and gene expression between 2D and 3D cell cultures, multiple paired *t*-tests, corrected for multiple corrections using the Holm–Šídák method, were used for each of the included analyses. For comparison between each treatment group and the untreated control (Ctrl) within 2D or 3D cell cultures, one-way analysis of variance (ANOVA) with Dunnett’s multiple comparison correction was used. Comparisons between 2D and 3D cell cultures (significances indicated by *) as well as between each treatment group (significances indicated by #) were made for all proteins and genes included in the analysis. Data are presented as the mean ± standard deviation (SD). No prior power calculation was conducted, and *n* = 3–5 experiments were performed depending on the specific method applied. A *p*-value < 0.05 was considered statistically significant after multiple correction.

## Figures and Tables

**Figure 1:**
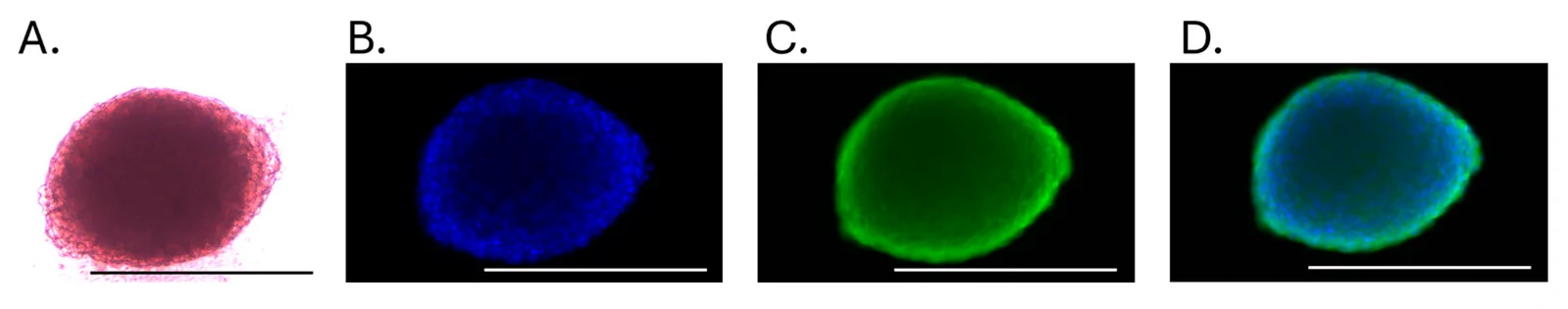
Spheroid of aortic smooth muscle cells. Imaging of three-dimensional culture of 15,000 aortic smooth muscle cells seeded in Nunclon™ Sphera™ 96-well U-bottom plate (Thermo Fisher Scientific). (**A**) Brightfield, (**B**) DAPI, (**C**) F-actin, (**D**) Merge. Detected by EVOS M5000 microscope (Thermo Fisher Scientific), scale bars = 400 µm.

**Figure 2:**
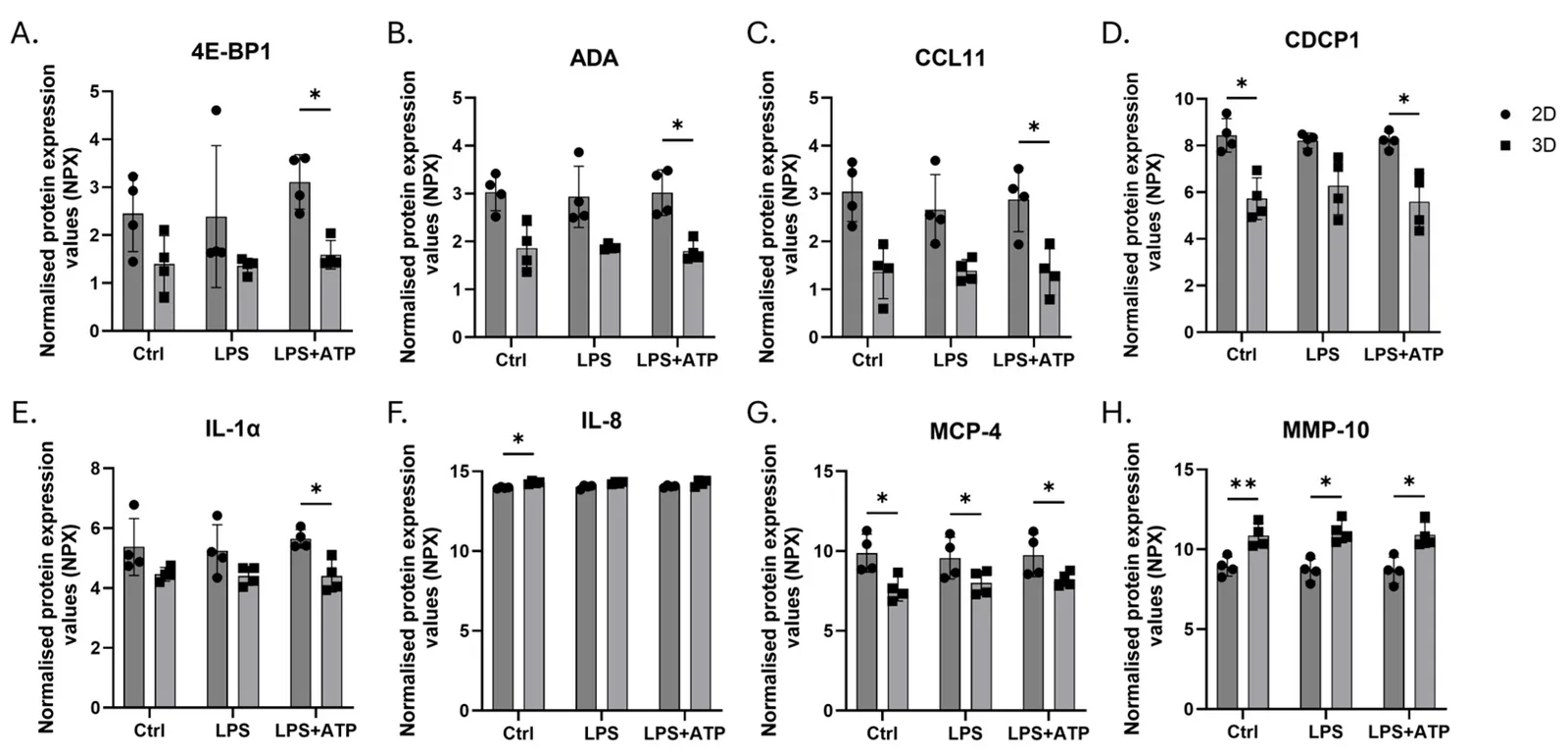
Differences in protein release between 2D and 3D cultured aortic smooth muscle cells consistent across all treatment conditions. Protein content in cell culture media after 24 h in presence or absence of LPS or LPS and ATP was detected by Olink Proteomics Inflammation panel. (**A**) Eukaryotic translation initiation factor 4E-binding protein 1 (4E-BP1), (**B**) adenosine deaminase (ADA), (**C**) eotaxin (CCL11), (**D**) CUB domain-containing protein 1 (CDCP1), (**E**) interleukin-1 alpha (IL-1α), (**F**) IL-8, (**G**) monocyte chemotactic protein 4 (MCP-4), (**H**) matrix metallopeptidase 10 (MMP-10). Data are shown as mean ± SD (*n* = 4), multiple paired T-test with Holm–Šídák multiple correction * *p* < 0.05, ** *p* < 0.01 was used to indicate significant differences in protein release between 2D and 3D cultured cells.

**Figure 3:**
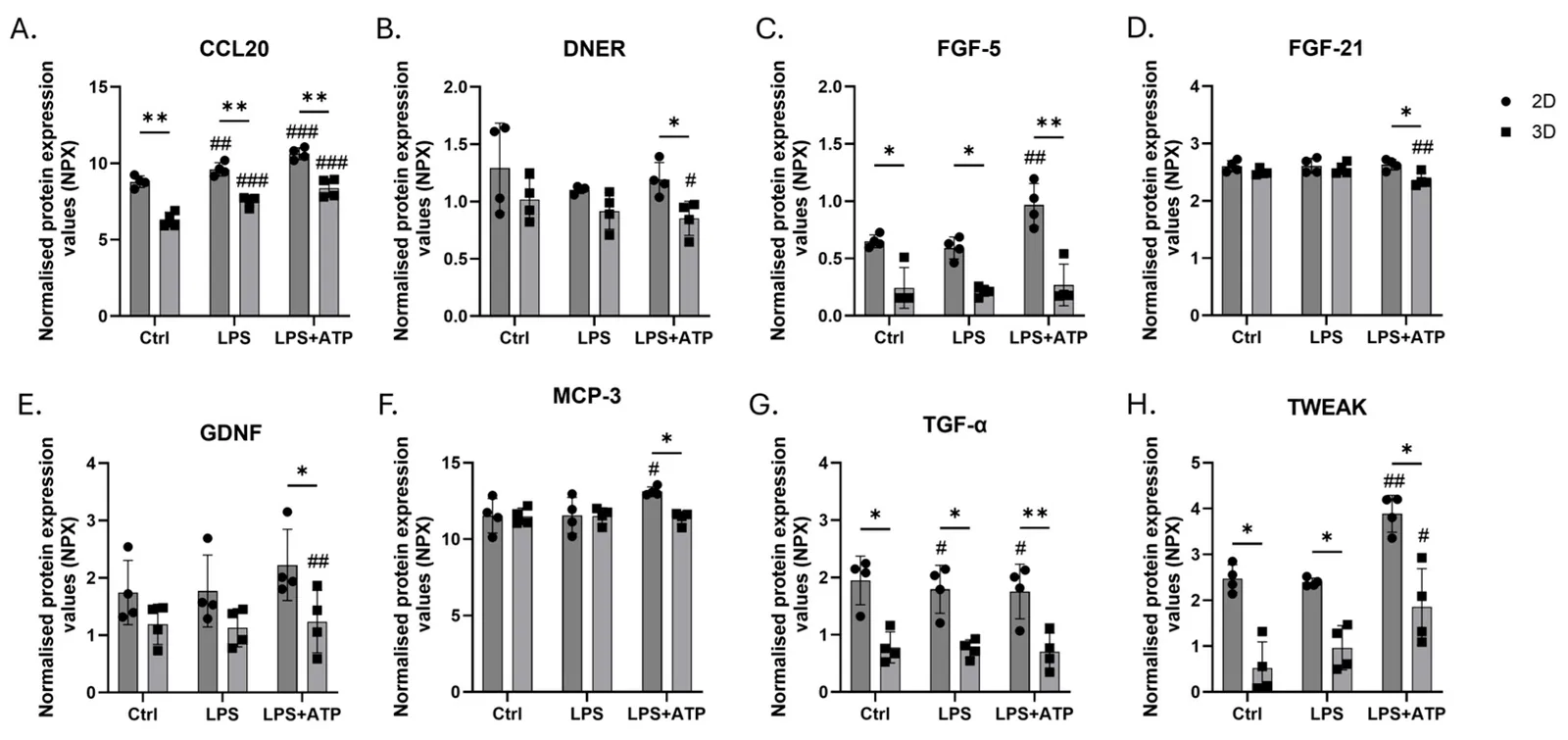
Differences in protein release in response to treatments and model of cultures of aortic smooth muscle cells. Protein content in cell culture media after 24 h treatment with LPS or LPS and ATP in 2D or 3D cell culture models were detected by Olink Proteomics Inflammation panel. (**A**) C-C motif chemokine 20 (CCL20), (**B**) Delta and Notch-like epidermal growth factor-related receptor (DNER), (**C**) fibroblast growth factor 5 (FGF-5), (**D**) FGF-21, (**E**) glial cell-derived neurotrophic factor (GDNF), (**F**) monocyte chemotactic protein 3 (MCP-3), (**G**) transforming growth factor alpha (TGFα), (**H**) tumor necrosis factor superfamily member 12 (TWEAK). Data are shown as mean ± SD (*n* = 4). Comparisons of treatments and untreated control within 2D or 3D cell cultures were made by one-way ANOVA with Dunnett’s multiple correction ^#^ *p* < 0.05, ^##^ *p* < 0.01, ^###^ *p* < 0.001. Multiple paired T-test with Holm–Šídák multiple correction * *p* < 0.05, ** *p* < 0.01 were used to compare between 2D and 3D cell culture models.

**Figure 4:**
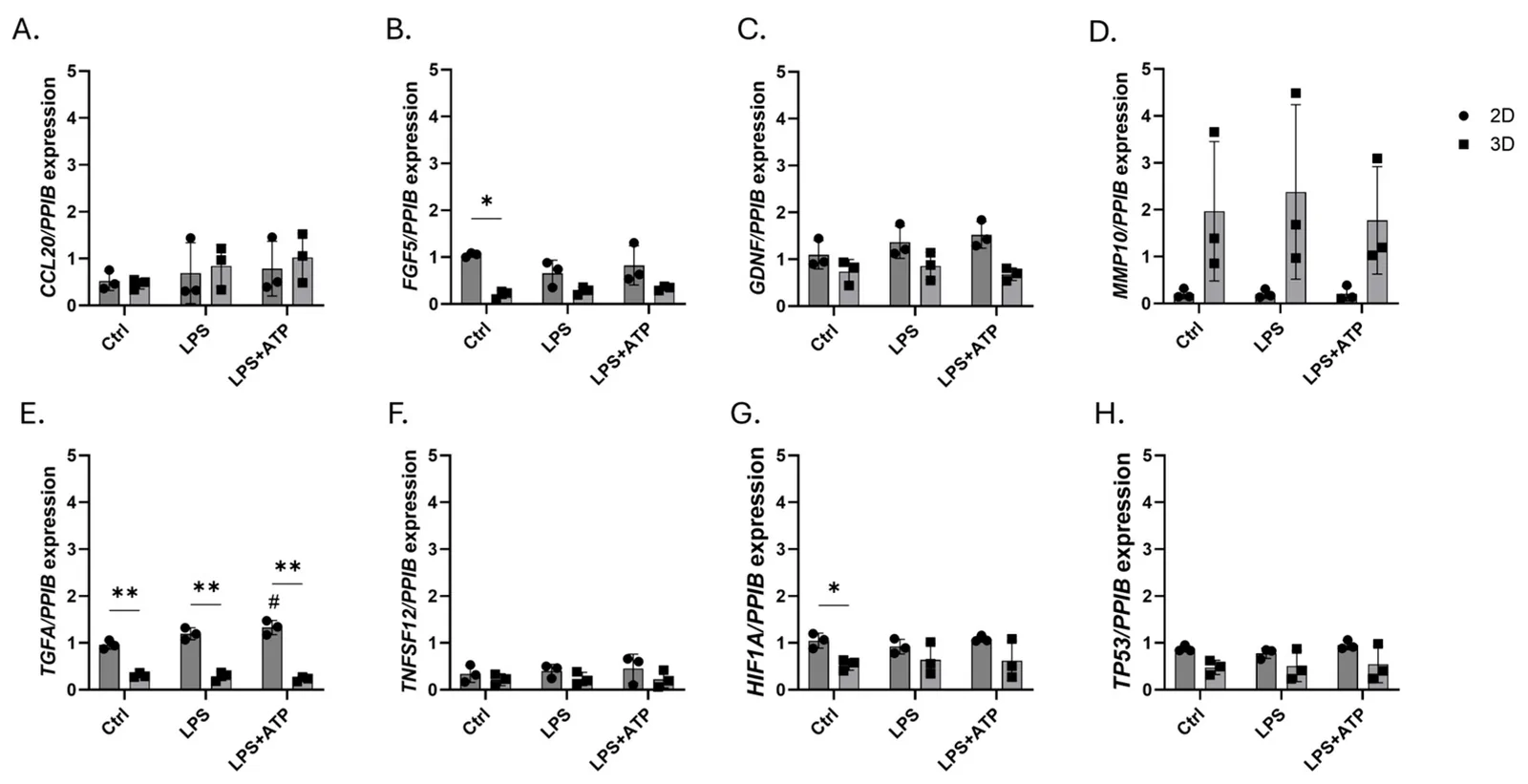
Alterations in gene expression between 2D and 3D cell cultures of aortic smooth muscle cells. Cultures of aortic smooth muscle cells in 2D or 3D format were treated with LPS or LPS and ATP for 24 h. Gene expression was measured by RT-qPCR of (**A**) *CCL20*, (**B**) *FGF5*, (**C**) *GDNF*, (**D**) *MMP10*, (**E**) *TGFA*, (**F**) *TNFSF12* (mRNA for TWEAK protein), (**G**) *HIF1A*, (**H**) *TP53*. Data are normalized to expression of *PPIB* and shown as mean ± SD (*n* = 3). Comparisons of treatments and untreated control within 2D or 3D cell cultures were made by one-way ANOVA with Dunnett’s multiple correction ^#^ *p* < 0.05. Multiple paired T-test with Holm–Šídák multiple correction * *p* < 0.05, ** *p* < 0.01 for were used to compare between 2D and 3D cell culture models.

**Figure 5:**
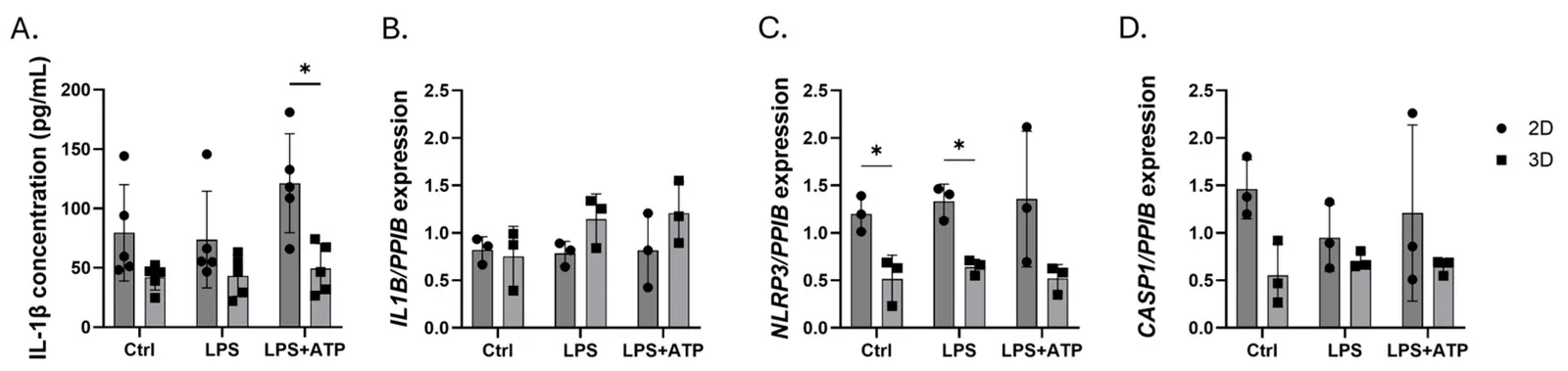
NLRP3 inflammasome and IL-1β in aortic smooth muscle cells. Cultures of aortic smooth muscle cells in 2D or 3D format were treated with LPS or LPS and ATP for 24 h. (**A**) Release of IL-1β measured by ELISA (*n* = 5). Gene expression was measured by RT-qPCR (*n* = 3) of (**B**) *IL1B*, (**C**) *NLRP3*, (**D**) *CASP1*. Data are normalized to expression of *PPIB* and shown as mean ± SD, multiple paired T-test with Holm–Šídák multiple correction * *p* < 0.05 were used to compare between 2D and 3D cell culture models.

## Data Availability

The original contributions presented in this study are included in the article/supplementary material. Further inquiries can be directed to the corresponding author.

## Supplementary Materials

The following supporting information can be downloaded at: https://www.mdpi.com/article/10.3390/ijms27198567/s1.

## Abbreviations

The following abbreviations are used in this manuscript:

2D	Two-dimensional
3D	Three-dimensional
4E-BP1	Eukaryotic translation initiation factor 4E-binding protein 1
ADA	Adenosine deaminase
ANOVA	Analysis of variance
AoSMC	Aortic smooth muscle cell
ATP	Adenosine triphosphate
CANTOS	Canakinumab Anti-inflammatory Thrombosis Outcome Study
*CASP1*	mRNA for Caspase-1
CCL	C-C motif chemokine
CCL11	Eotaxin
CDCP1	CUB domain-containing protein 1
Ctrl	Control
CVD	Cardiovascular disease
CXCL	C-X-C motif chemokine
DAMP	Danger associated molecular patterns
DAPI	4′,6-diamidino-2-phenylindole
DNER	Delta and Notch-like epidermal growth factor-related receptor
ECM	Extracellular matrix
FGF	Fibroblast growth factor
GDNF	Glial cell-derived neurotrophic factor
IL	Interleukin
LAP TGF-β-1	Transforming growth factor beta-1 proprotein
LPS	Lipopolysaccharide
MCP	Monocyte chemoattractant protein
MMP	Matrix metallopeptidase
NF-κB	Nuclear factor kappa-light-chain-enhancer of activated B cells
NPX	Normalised protein expression levels
NRTN	Neurturin
TGF	Transforming growth factor
TLR	Toll-like receptor
TNF	Tumor necrosis factor
*TNFSF12*	mRNA for TWEAK protein
TWEAK	TNF superfamily member 12
PAMP	Pathogen associated molecular patterns
PCR	Polymerase chain reaction
PEA	Proximity Extension Assay
PEST	Penicillin-streptomycin
PPIB	Peptidyl-prolyl-cis-trans isomerase B
SD	Standard deviation
uPA	Urokinase-type plasminogen activator

## References

[1] Martin S.S., Aday A.W., Allen N.B., Almarzooq Z.I., Anderson C.A.M., Arora P., Avery C.L., Baker-Smith C.M., Bansal N., Beaton A.Z. (2025). 2025 Heart Disease and Stroke Statistics: A Report of US and Global Data From the American Heart Association. Circulation.

[2] Libby P., Ridker P.M., Hansson G.K. (2011). Progress and challenges in translating the biology of atherosclerosis. Nature.

[3] Libby P. (2021). The changing landscape of atherosclerosis. Nature.

[4] Frostegard J. (2013). Immunity, atherosclerosis and cardiovascular disease. BMC Med..

[5] Ridker P.M., Everett B.M., Thuren T., MacFadyen J.G., Chang W.H., Ballantyne C., Fonseca F., Nicolau J., Koenig W., Anker S.D. (2017). Antiinflammatory Therapy with Canakinumab for Atherosclerotic Disease. N. Engl. J. Med..

[6] Mazzoleni G., Di Lorenzo D., Steimberg N. (2009). Modelling tissues in 3D: The next future of pharmaco-toxicology and food research?. Genes Nutr..

[7] Duval K., Grover H., Han L., Mou Y., Pegoraro A.F., Fredberg J., Chen Z. (2017). Modeling Physiological Events in 2D vs. 3D Cell Culture. Physiology.

[8] Bhadriraju K., Chen C.S. (2002). Engineering cellular microenvironments to improve cell-based drug testing. Drug Discov. Today..

[9] Cukierman E., Pankov R., Stevens D.R., Yamada K.M. (2001). Taking cell-matrix adhesions to the third dimension. Science.

[10] Li H., Zhou H., Xu C., Wei Y., Tang X. (2023). 3D bioprinting and scaffold-free strategies for fabrication of multi-cellular tissues or organoids. Int. J. Bioprint..

[11] Zhang L., Liao W., Chen S., Chen Y., Cheng P., Lu X., Ma Y. (2023). Towards a New 3Rs Era in the construction of 3D cell culture models simulating tumor microenvironment. Front. Oncol..

[12] Bialkowska K., Komorowski P., Bryszewska M., Milowska K. (2020). Spheroids as a Type of Three-Dimensional Cell Cultures—Examples of Methods of Preparation and the Most Important Application. Int. J. Mol. Sci..

[13] Arulselvan P., Fard M.T., Tan W.S., Gothai S., Fakurazi S., Norhaizan M.E., Kumar S.S. (2016). Role of Antioxidants and Natural Products in Inflammation. Oxid. Med. Cell Longev..

[14] Frismantiene A., Philippova M., Erne P., Resink T.J. (2018). Smooth muscle cell-driven vascular diseases and molecular mechanisms of VSMC plasticity. Cell Signal..

[15] Chen J., Zhang X., Millican R., Lynd T., Gangasani M., Malhotra S., Sherwood J., Hwang P.T., Cho Y., Brott B.C. (2022). Recent Progress in in vitro Models for Atherosclerosis Studies. Front. Cardiovasc. Med..

[16] Biju T.S., Veeraraghavan V.P., Francis A.P. (2023). Role of three-dimensional cell culture in therapeutics and diagnostics: An updated review. Drug Deliv. Transl. Res..

[17] Al-Lamki R.S., Bradley J.R., Pober J.S. (2017). Human Organ Culture: Updating the Approach to Bridge the Gap from In Vitro to In Vivo in Inflammation, Cancer, and Stem Cell Biology. Front. Med..

[18] Azar P., Jarr K.U., Gomez D., Jørgensen H.F., Leeper N.J., Bochaton-Piallat M.L. (2025). Smooth muscle cells in atherosclerosis: Essential but overlooked translational perspectives. Eur. Heart J..

[19] Zernecke A., Weber C. (2014). Chemokines in atherosclerosis: Proceedings resumed. Arterioscler. Thromb. Vasc. Biol..

[20] Hou X.Z., Yang Y.T., Yao J.M. (2026). Vulnerable plaques in atherosclerosis: Focus on angiogenesis-associated phenotypic crosstalk. Front. Pharmacol..

[21] Mauersberger C., Hinterdobler J., Schunkert H., Kessler T., Sager H.B. (2022). Where the Action Is-Leukocyte Recruitment in Atherosclerosis. Front. Cardiovasc. Med..

[22] Dautova Y., Kapustin A.N., Pappert K., Epple M., Okkenhaug H., Cook S.J., Shanahan C.M., Bootman M.D., Proudfoot D. (2018). Calcium phosphate particles stimulate interleukin-1β release from human vascular smooth muscle cells: A role for spleen tyrosine kinase and exosome release. J. Mol. Cell Cardiol..

[23] Jäger M.A., De La Torre C., Arnold C., Kohlhaas J., Kappert L., Hecker M., Feldner A., Korff T. (2020). Assembly of vascular smooth muscle cells in 3D aggregates provokes cellular quiescence. Exp. Cell Res..

[24] Paramel G.V., Lindkvist M., Idosa B.A., Sebina L.S., Kardeby C., Fotopoulou T., Pournara D., Kritsi E., Ifanti E., Zervou M. (2022). Novel purine analogues regulate IL-1β release via inhibition of JAK activity in human aortic smooth muscle cells. Eur. J. Pharmacol..

[25] Aherrahrou R., Baig F., Theofilatos K., Lue D., Beele A., Örd T., Kaikkonen M.U., Aherrahrou Z., Cheng Q., Ghosh S.K.B. (2024). Secreted Protein Profiling of Human Aortic Smooth Muscle Cells Identifies Vascular Disease Associations. Arterioscler. Thromb. Vasc. Biol..

[26] Alpert T., Herzel L., Neugebauer K.M. (2017). Perfect timing: Splicing and transcription rates in living cells. Wiley Interdiscip. Rev. RNA.

[27] Kudelski J., Młynarczyk G., Gudowska-Sawczuk M., Mroczko B., Darewicz B., Bruczko-Goralewska M., Romanowicz L. (2022). Higher Content but Not Activity of Stromelysin-2 (MMP-10) in Comparison to Stromelysin-1 (MMP-3) in Human Renal Carcinoma. Int. J. Environ. Res. Public Health.

[28] Martinez-Aguilar E., Gomez-Rodriguez V., Orbe J., Rodriguez J.A., Fernández-Alonso L., Roncal C., Páramo J.A. (2015). Matrix metalloproteinase 10 is associated with disease severity and mortality in patients with peripheral arterial disease. J. Vasc. Surg..

[29] Rodriguez J.A., Orbe J., Martinez de Lizarrondo S., Calvayrac O., Rodriguez C., Martinez-Gonzalez J., Paramo J.A. (2008). Metalloproteinases and atherothrombosis: MMP-10 mediates vascular remodeling promoted by inflammatory stimuli. Front. Biosci..

[30] Mishra D.K., Sakamoto J.H., Thrall M.J., Baird B.N., Blackmon S.H., Ferrari M., Kurie J.M., Kim M.P. (2012). Human lung cancer cells grown in an ex vivo 3D lung model produce matrix metalloproteinases not produced in 2D culture. PLoS ONE.

[31] Rohani M.G., McMahan R.S., Razumova M.V., Hertz A.L., Cieslewicz M., Pun S.H., Regnier M., Wang Y., Birkland T.P., Parks W.C. (2015). MMP-10 Regulates Collagenolytic Activity of Alternatively Activated Resident Macrophages. J. Investig. Dermatol..

[32] Karamanos N.K., Theocharis A.D., Piperigkou Z., Manou D., Passi A., Skandalis S.S., Vynios D.H., Orian-Rousseau V., Ricard-Blum S., Schmelzer C.E.H. (2021). A guide to the composition and functions of the extracellular matrix. FEBS J..

[33] Saraswathibhatla A., Indana D., Chaudhuri O. (2023). Cell–extracellular matrix mechanotransduction in 3D. Nat. Rev. Mol. Cell Biol..

[34] Afonina I.S., Zhong Z., Karin M., Beyaert R. (2017). Limiting inflammation-the negative regulation of NF-κB and the NLRP3 inflammasome. Nat. Immunol..

[35] Björkerud S. (1987). Agglomeration to nodules modulates human arterial smooth muscle cells to distinct postinjury phenotype via foam cell transition. Am. J. Pathol..

[36] Ross R. (1971). The smooth muscle cell. II. Growth of smooth muscle in culture and formation of elastic fibers. J. Cell Biol..

[37] Abuwatfa W.H., Pitt W.G., Husseini G.A. (2024). Scaffold-based 3D cell culture models in cancer research. J. Biomed. Sci..

[38] Mittler F., Obeïd P., Rulina A.V., Haguet V., Gidrol X., Balakirev M.Y. (2017). High-Content Monitoring of Drug Effects in a 3D Spheroid Model. Front. Oncol..

[39] Yu M.H., Li X., Li Q., Mo S.J., Ni Y., Han F., Wang Y.B., Tu Y.X. (2019). SAA1 increases NOX4/ROS production to promote LPS-induced inflammation in vascular smooth muscle cells through activating p38MAPK/NF-κB pathway. BMC Mol. Cell Biol..

[40] Tweedell R.E., Malireddi R.K.S., Kanneganti T.D. (2020). A comprehensive guide to studying inflammasome activation and cell death. Nat. Protoc..

