# Supplementary material for: Altered Inflammatory Protein Release by Vascular Smooth Muscle Cells in 3D-Culture
Source: Int J Mol Sci. 2026 Sep 25;27(19):8567. doi: 10.3390/ijms27198567 (PMC13665300; doi:10.3390/ijms27198567)
Supplement: Supplementary file 1 [file ijms-27-08567-s001.zip › ijms-4447008-supplementary.pdf]

Supplementary data

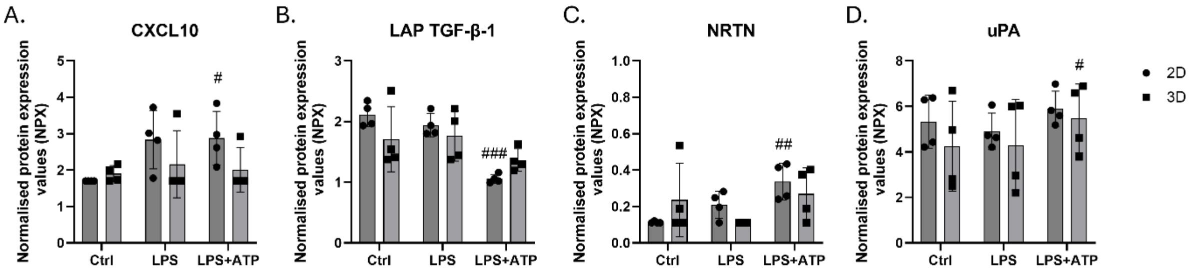

Figure S1. Up- and downregulated protein releases from aortic smooth muscle cells in response to treatment. Protein content in cell culture media after 24 hours treatment with LPS or LPS and ATP was detected by Olink Proteomics Inflammatory panel. Release of **A.** C-X-C motif chemokine 10 (CXCL10), **B.** Transforming growth factor beta-1 proprotein (LAP TGF-β-1), **C.** Neurturin (NRTN) and **D.** Urokinase-type plasminogen activator (uPA) were significantly different after LPS and ATP treatment compared to the corresponding untreated control within the group of 2D or 3D culture. Data are shown as mean±SD (n=4), one-way ANOVA with Dunnett's multiple correction $^{\#}p<0.05$, $^{\#\#}p<0.01$, $^{\#\#\#}p<0.001$.

Table S1. Included proteins from Olink Target 96 Inflammation Panel. Out of the 92 protein assays in the Olink Target 96 Inflammation Panel, 47 were included in the statistical analysis. Exclusion criteria was a missing data frequency >50%.

Included assays	Full name	Excluded assays	Full name
4E-BP1	Eukaryotic translation initiation factor 4E-binding protein 1	ARTN	Artemin
ADA	Adenosine deaminase	Beta-NGF	Beta-nerve growth factor
AXIN1	Axin-1	CCL23	C-C motif chemokine 23
CASP-8	Caspase-8	CCL25	C-C motif chemokine 25
CCL3	C-C motif chemokine 3	CCL28	C-C motif chemokine 28
CCL4	C-C motif chemokine 4	CD5	T-cell surface glycoprotein CD5
CCL11	C-C motif chemokine 11 (eotaxin)	CD6	T-cell differentiation antigen CD6
CCL19	C-C motif chemokine 19	CD8α	T-cell surface glycoprotein CD8 alpha chain
CCL20	C-C motif chemokine 20	CD244	Natural killer cell receptor 2B4
CD40	CD40 molecule	CST5	Cystatin D
CDCP1	CUB domain-containing protein 1	CX3CL1	Fractalkine
CSF-1	Colony stimulating factor 1	CXCL9	C-X-C motif chemokine 9
CXCL1	C-X-C motif chemokine ligand 1	CXCL11	C-X-C motif chemokine 11
CXCL5	C-X-C motif chemokine ligand 5	EN-RAGE	Protein S100-A12
CXCL6	C-X-C motif chemokine ligand 6	FGF-23	Fibroblast growth factor 23
CXCL10	C-X-C motif chemokine ligand 10	FGF-19	Fibroblast growth factor 19
DNER	Delta and Notch-like epidermal growth factor-related receptor	IFNγ	Interferon gamma
FGF-5	Fibroblast growth factor 5	IL-2	Interleukin-2
FGF-21	Fibroblast growth factor 21	IL-2RB	Interleukin-2 receptor subunit beta
Flt3L	Fms related receptor tyrosine kinase 3 ligand	IL-4	Interleukin-4
GDNF	Glial cell derived neurotrophic factor	IL-5	Interleukin-5
HGF	Hepatocyte growth factor	IL-7	Interleukin-7
IL-1α	Interleukin-1 alpha	IL-10	Interleukin-10
IL-6	Interleukin-6	IL-10Rα	Interleukin-10 receptor subunit alpha
IL-8	Interleukin-8	IL-10Rβ	Interleukin-10 receptor subunit beta
IL-12β	Interleukin-12 subunit beta	IL-13	Interleukin-13
IL-24	Interleukin-24	IL-15Rα	Interleukin-15 receptor subunit alpha
IL-33	Interleukin-33	IL-17A	Interleukin-17A
LAP TGF-β-1	Transforming growth factor beta-1 proprotein	IL-17C	Interleukin-17C
LIF	Leukemia inhibitory factor	IL-18	Interleukin-18
MCP-1	Monocyte chemotactic protein 1	IL-18R1	Interleukin-18 receptor 1
MCP-2	Monocyte chemotactic protein 2	IL-20	Interleukin-20
MCP-3	Monocyte chemotactic protein 3	IL-20Rα	Interleukin-20 receptor subunit alpha
MCP-4	Monocyte chemotactic protein 4	IL-22Rα1	Interleukin-22 receptor subunit alpha-1
MMP-1	Matrix metallopeptidase 1	LIF-R	Leukemia inhibitory factor receptor
MMP-10	Matrix metallopeptidase 10	NT-3	Neurotrophin-3
NRTN	Neurturin	OSM	Oncostatin-M
OPG	Osteoprotegerin	SLAMF1	Signaling lymphocytic activation molecule family member 1
PD-L1	Programmed cell death 1 ligand 1	ST1A1	Sulfotransferase family 1A member 1
SCF	Stem cell factor	TNF	Tumor necrosis factor
SIRT2	Sirtuin 2	TNFβ	Lymphotoxin-alpha (tumor necrosis factor-beta)
STAMBP	STAM-binding protein	TNFRSF9	Tumor necrosis factor receptor superfamily member 9
TGF-α	Transforming growth factor alpha	TNFSF14	Tumor necrosis factor ligand superfamily member 14
TSLP	Thymic stromal lymphopoietin	TRAIL	Tumor necrosis factor-related apoptosis-inducing ligand
TWEAK	Tumor necrosis factor superfamily member 12	TRANCE	Tumor necrosis factor-related activation-induced cytokine
uPA	Urokinase-type plasminogen activator		
VEGFA	Vascular endothelial growth factor A		
